# Outcome of immune checkpoint inhibitors in patients with extensive-stage small-cell lung cancer and brain metastases

**DOI:** 10.3389/fonc.2023.1110949

**Published:** 2023-05-05

**Authors:** Chunyu Wang, Shuai Mu, Xuhui Yang, Lingling Li, Haitao Tao, Fan Zhang, Ruixin Li, Yi Hu, Lijie Wang

**Affiliations:** ^1^ Department of Oncology, The First Medical Center of Chinese People’s Liberation Army (PLA) General Hospital, Beijing, China; ^2^ Department of Oncology, The Fifth Medical Center of Chinese People’s Liberation Army (PLA) General Hospital, Beijing, China; ^3^ Department of Oncology, Chinese PLA Medical School, Beijing, China

**Keywords:** extensive stage small-cell lung cancer, brain metastases, immune checkpoint inhibitor, progression-free survival, the intracranial failure

## Abstract

**Objectives:**

Brain metastases (BMs) are common in extensive-stage small-cell lung cancer (SCLC) and are underrepresented in pivotal clinical trials that demonstrate the efficacy of immune checkpoint inhibitors (ICIs). We conducted a retrospective analysis to assess the role of ICIs in BM lesions in less selected patients.

**Materials and methods:**

Patients with histologically confirmed extensive-stage SCLC who were treated with ICIs were included in this study. Objective response rates (ORRs) were compared between the with-BM and without-BM groups. Kaplan−Meier analysis and the log-rank test were used to evaluate and compare progression-free survival (PFS). The intracranial progression rate was estimated using the Fine-Gray competing risks model.

**Results:**

A total of 133 patients were included, 45 of whom started ICI treatment with BMs. In the whole cohort, the overall ORR was not significantly different for patients with and without BMs (p = 0.856). The median progression-free survival for patients with and without BMs was 6.43 months (95% CI: 4.70-8.17) and 4.37 months (95% CI: 3.71-5.04), respectively (p =0.054). In multivariate analysis, BM status was not associated with poorer PFS (p = 0.101). Our data showed that different failure patterns occurred between groups, with 7 patients (8.0%) without BM and 7 patients (15.6%) with BM having intracranial-only failure as the first site progression. The cumulative incidences of brain metastases at 6 and 12 months were 15.0% and 32.9% in the without-BM group and 46.2% and 59.0% in the BM group, respectively (Gray’s p<0.0001).

**Conclusions:**

Although patients with BMs had a higher intracranial progression rate than patients without BMs, the presence of BMs was not significantly associated with a poorer ORR and PFS with ICI treatment in multivariate analysis.

## 1 Introduction

Small-cell lung cancer (SCLC) is the most common type of neuroendocrine tumor and accounts for approximately 14% of lung cancers (1, 2). Approximately two-thirds of SCLC patients present with extensive-stage disease (ES-SCLC) (3). Brain metastases (BMs) occur in more than 50% of patients with extensive SCLC (4). Despite this high incidence, only patients with treated and/or asymptomatic BMs have been eligible for first-line immune checkpoint inhibitor (ICI) clinical trials (5–7). Therefore, patients with BMs, ranging from 8.7% to 12.1%, were highly underrepresented in these guideline-changing clinical trials that used ICIs as second and further lines of treatment for ES-SCLC patients (8, 9).

In addition, few clinical trials had a planned subgroup analysis based on BMs. IMpower133 and CASPIAN, randomized phase III clinical trials, showed that the addition of a programmed cell death ligand 1 (PD-L1) antibody to chemotherapy benefited the overall survival of ES-SCLC patients; however, in the BM subgroup, the patients taking ICIs did not exhibit survival superiority. The same results were observed in the KEYNOTE-604 trial, which used a programmed cell death 1 (PD-1) inhibitor (5–7). Furthermore, in the aforementioned clinical trials, the failure patterns were not reported, which are crucial data for doctors in making further treatment recommendations, such as thoracic radiotherapy or prophylactic cranial irradiation (PCI).

To the best of our knowledge, there are neither prospective clinical trials to estimate the role of ICIs in BM patients nor retrospective trials that analyze the response of BMs to ICIs due to the short time ICIs have been approved for treatment in SCLC.

Therefore, in this retrospective study, we focused on comparing the outcome of the less selected extensive-stage SCLC patients with BMs treated with ICIs to patients without BMs. Second, we demonstrated the failure pattern of patients, especially intracranial failure, to provide more information for further decisions on local treatment.

## 2 Materials and methods

### 2.1 Patients

The institutional review board at our institution approved the present study. We retrospectively reviewed SCLC patients treated at our institution from January 2015 to December 2020. All patients included in this study met the following criteria: 1) histologically or cytologically confirmed SCLC; 2) extensive-stage SCLC as defined by the Veterans Administration Lung Study Group staging system; 3) measurable extensive-stage small-cell lung cancer according to Response Evaluation Criteria in Solid Tumors (RECIST 1.1) (10); 4) treatment with PD-1/PD-L1 for at least 2 cycles or one cycle with image review; and 5) complete pretreatment baseline data and follow-up data.

The exclusion criteria included the following: 1) limited-stage SCLC; 2) MRI-confirmed leptomeningeal metastasis; 3) use of PD-1/PD-L1 inhibitors as consolidative treatment; 4) no follow-up data available; 5) use of other immune-related treatment, including anti-CTLA-4 treatment and cellular immunotherapy; and 6) synchronous or metachronous malignancies (except for cutaneous (nonmelanoma) carcinoma, thyroid papillary carcinoma, phase I seminoma or cervical carcinoma *in situ* that were curatively treated).

The patients were treated with the following regimens: 1) for patients who were treatment-naive: patients received etoposide and cisplatin/carboplatin and PD-1/PD-L1 as first-line therapy; 2) for patients who failed on previous chemotherapy: patients received PD-1/PD-L1 agents as second-line treatment and beyond, a single PD-1/PD-L1 agent or a combined PD-1/PD-L1 with chemotherapy (usually irinotecan) were given depending on the choice of the medical oncologists. The patients would receive the brain radiotherapy concurrently or subsequently with ICI depending on the doctor’s decision.

The following variables were reviewed for analyses: date of birth, sex, smoking history, Karnofsky Performance Score (KPS) when PD-1/PD-L1 inhibitors treatment was started, BM diagnosis date, number of brain metastases, maximum size of BMs, presence of BM symptoms, extracranial metastases status, and name of PD-1/PD-L1 treatment. The disease-specific graded prognostic assessment (ds-GPA) was calculated according to a published study (11).

Patients who had at least one brain metastasis larger than 5 mm that was untreated or unequivocally progressed after radiation before the start of ICI treatment were defined as having active BMs [revised from (12)]. Brain radiation was not mandatory for this group of patients given some of them had brain radiation previously or without symptoms. Stable BMs were defined as those that had been treated with radiotherapy or surgery before ICI treatment and showed no progression on brain imaging no more than 6 weeks before the start of ICI treatment [based on (13)]. The type of brain radiation was given to patients according to the choice of radiation oncologists.

The intracranial lesions were evaluated every two to three months by magnetic resonance imaging (MRI) or computed tomography (CT) with contrast, and the primary lesion and other metastasis sites were monitored by CT or positron emission tomography–CT if needed every two to three months. If a patient lost follow up, we censored the data on the date that was accurate. The objective response rate (ORR) and disease control rate (DCR) were evaluated using the Response Evaluation Criteria in Solid Tumors (RECIST, version 1.1) (10). The first progression site (intracranial, extracranial or both) and date were recorded. The most recent follow-up time was recorded.

### 2.2 Statistical analysis

The patient characteristics, ORR, and DCR in both groups were compared with the χ^2^ test or Fisher’s exact test for categorical variables and one-way analysis of variance for continuous data. PFS was calculated from the date of PD-1/PD-L1 initiation to the date of objective disease progression or death from any cause in the absence of progression. OS was derived from the date of PD-1/PD-L1 treatment until the date of death or censored on the last follow-up. Kaplan−Meier analysis was used to estimate PFS and OS. The log-rank test was used to compare the data.

Considering the competing risk of death to intracranial progression, we used Fine-Gray competing risk regression to compare the cumulative incidence rate of intracranial recurrence (14).

Univariable and multivariable Cox proportional hazards analyses examined factors associated with an increased risk of progression and death. Significance for inclusion in the multivariate model was set at p < 0.10, and p < 0.05 was set as the significance level for predictors of outcomes. Statistical analyses were performed using R (Version 4.1.2).

## 3 Results

### 3.1 Patient selection and characteristics

**Figure 1:**
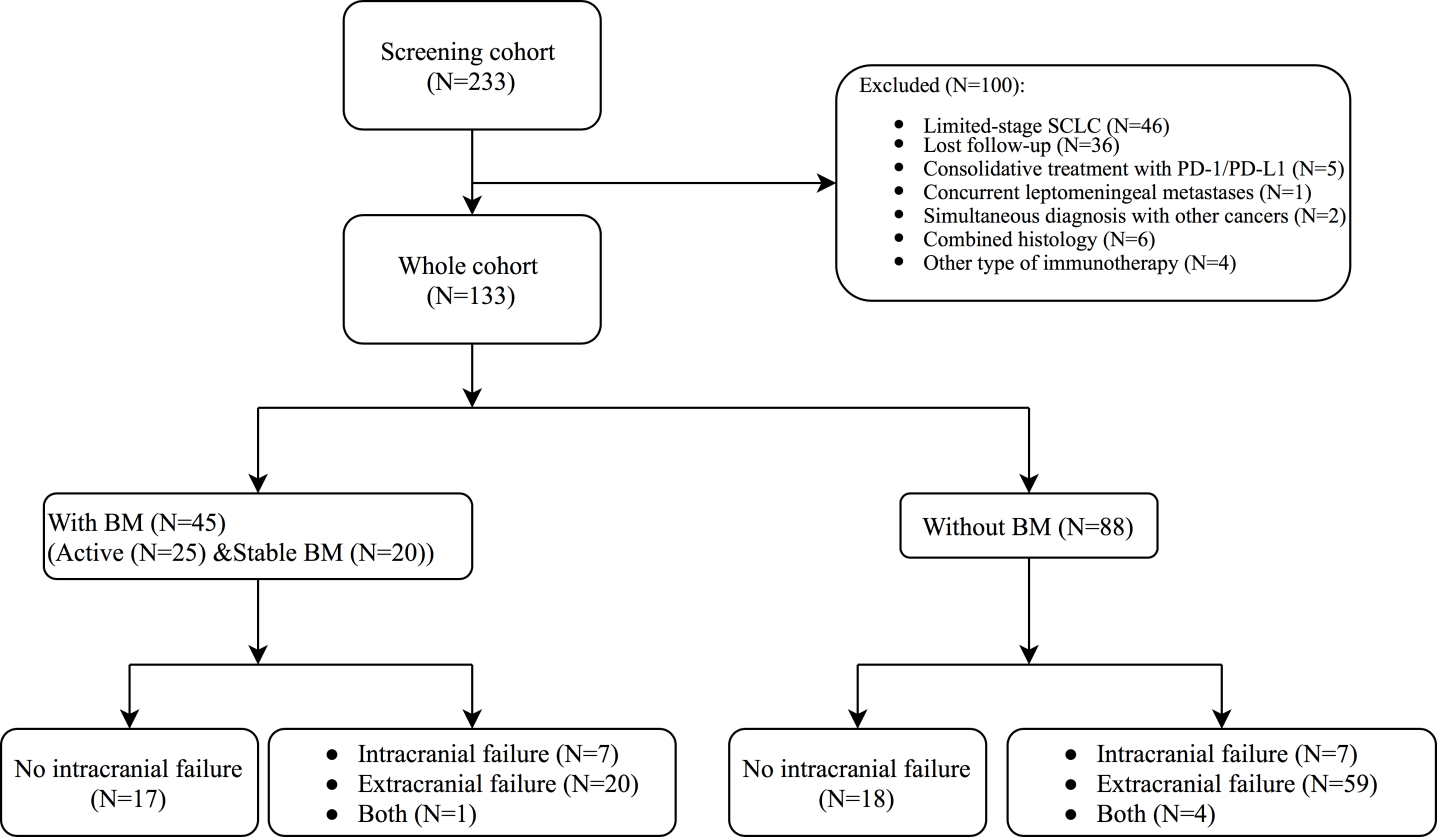
The enrollment and Outcomes of the Patients. BM, brain metastases.

Between January 1^st^, 2015, and December 31^st^, 2020, data on 233 patients were collected. Of these patients, 46 patients were excluded because they had limited-stage SCLC, and 36 patients were excluded because they were lost to follow-up after the first dose of PD-1/PD-L1 treatment without subsequent image review. Five patients were excluded due to consolidative treatment with PD-1/PD-L1. One patient was excluded due to concurrent leptomeningeal metastases; two patients were excluded due to simultaneous diagnosis with other cancers; six patients had combined histology; and four patients received NK-cell immunotherapy before or after PD-1/PD-L1 treatment. The remaining 133 patients were included, 45 (33.8%) of whom had brain metastases (**Figure 1**). Baseline characteristics are presented in **Table 1** according to BM status.

**Table 1:** Patient characteristics.

	Total Population (N=133) (%)	Patients without Baseline Brain Metastases (N=88) (%)	Patients with Baseline Brain Metastases (N=45) (%)	*p*
Age at BM (years)				
>60	64 (48.1)	49 (55.7)	15 (33.3)	0.05
50-60	55 (41.4)	31 (35.2)	24 (53.3)	
<50	14 (10.5)	8 (9.1)	6 (13.3)	
Gender				
Male	114 (85.7)	75 (85.2)	39 (86.7)	1.00
Female	19 (14.3)	13 (14.8)	6 (13.3)	
KPS				
<70	1 (0.8)	0 (0)	1 (2.2)	0.28
70-80	54 (40.6)	38 (43.2)	16 (35.6)	
90-100	78 (58.6)	50 (56.8)	28 (62.2)	
Smoking history				
No	31 (23.3)	21 (23.9)	10 (22.2)	1.00
Yes	102 (76.7)	67 (76.1)	35 (77.8)	
No. of organs with extracranial metastases at start of treatment				
0	14 (10.5)	0 (0)	14 (31.1)	0.000
1	43 (32.2)	32 (36.4)	11 (24.4)	
2-6	76 (57.1)	56 (63.6)	20 (44.4)	
PD-1/PD-L1 inhibitor				
PD-1	104 (78.2)	67 (76.1)	37 (82.2)	0.51
PD-L1	29 (21.8)	21 (23.9)	8 (17.8)	
Line of ICIs treatment				
1	56 (42.1)	41 (46.6)	15 (33.3)	0.36
2	41 (30.8)	25 (28.4)	16 (35.6)	
Over 2	36 (27.1)	22 (25.0)	14 (31.1)	
Cycles of ICIs (median, range)	4(1-26)	4 (1-26)	4 (1-20)	0.45
Thoracic RT before start of ICIs treatment				
No	84 (63.2)	65 (73.9)	19 (42.2)	0.001
Yes	49 (36.8)	23 (26.1)	26 (57.8)	
Brain RT before start of ICIs treatment				
No	104 (68.2)	83 (94.3)	21 (46.7)	0.00
Yes	29 (21.8)	5 (5.7)	24 (53.3)	

KPS, Karnofsky Performance Score; BM, brain metastasis; RT, radiation therapy; ICIs, immune checkpoint inhibitors; PD-1, programmed cell death 1; PD-L1, programmed cell death ligand 1.

Most patients were smokers (76.7%) and male (85.7%). Patients without BMs had a heavier extracranial tumor burden; 63.6% (56/88) of patients without BMs had more than one metastasis, and 31.1% of patients in the BM group did not have extracranial metastases. The patients without BMs were older than the patients with BMs, and fewer patients received thoracic radiation (26.1% vs. 57.8%) before ICI treatment. Five (5.7%) patients in without BMs received PCI before the PD-1/PD-L1 initiation. In the BM group, 24 (53.3%) received brain radiation before ICI treatment including 23 of them had whole brain radiation (3 of them had boost radiation dose to the brain metastases gross tumor) and one patient had gamma knife to the brain metastases. There was no difference between the two groups in sex, KPS, ICI type or line of ICI treatment. Patients received PD-1/PD-L1 agents depending on the access to the drugs or the clinical trials, including durvalumab, pembrolizumab, atezolizuman, nivolumab, Sintilimab and toripalimab.

Details on the patients with BMs are shown in **Supplementary Table 1**. In all, 25 patients had active brain metastases, and 20 patients had stable metastases. The ds-GPA classification was 0 to 1 in 10 patients (22.2%), 1.5 to 2.0 in 19 patients (42.2%), 2.5 to 3.0 in 12 patients (26.7%), and 3.5 to 4.0 in 4 patients (8.9%). Two patients (4.4%) had symptomatic BMs at the start of ICI treatment. There was no difference between the two groups in smoking history, sex, ICI type, largest size of BM, ds-GPA score or symptoms from BM. Patients in the stable group had less BM number than those in the active group (median 2 vs. 4). Ninety percent (18/20) of patients in the stable group received radiation (all of it done previously), 72% of people with active BM had radiation (24% (6/25) previously and 52% (13/25) concurrently) given one patient had PCI before ICI and had SRS to the BM nodule with ICI.

In the active BM group, 12 (48.0%) out of 25 patients had concurrent brain radiotherapy while no patients had brain radiotherapy in the stable BM group.

Five patients received dexamethasone prior to ICIs due to symptomatic BMs (N=1), post surgery of brain metasteses (N=1) and the clinicians’ decision (N=3).

### 3.2 Treatment outcome

#### 3.2.1 Responses

In the whole cohort, the overall ORR was not significantly different for patients with and without BMs (46.7% (21/45) versus 48.9% (43/88); *p* = 0.856), and the DCR was lower but not significant in patients with BMs (73.3% (33/45) versus 83.0% (73/88); *p* =0.265). In patients who were treatment-naive, the ORR for the ICI combined regimen was 80.0% (12/15) in the patients with BMs and 80.5% (33/41) in the patients without BMs (*p*=1.000). The DCRs were 93.3% (14/15) and 95.1% (39/41), respectively (*p*=1.000).

Of the 45 patients with BMs, patients with active BMs had a not significantly different ORR as those with stable BMs (56.0% vs. 35.0%; *p*=0.231) and the same DCR (76.0% vs. 70.0%, *p*=0.741). In total, five patients had dissociated intracranial and extracranial responses. One patient had brain-only progressive disease (PD) with an extracranial response, and six patients had extracranial PD with intracranial stability. As to the five patients who received dexamethasone, three patients had partial response (PR) and two patients had progressive disease PD.

In our patients, we did not observe pseudoprogression in the brain. One patient experienced progression after the first dose of ICI treatment, and the progression was verified by subsequent image review.

#### 3.2.2 PFS and failure pattern

At the time of data cutoff, the median follow-up time was 17.63 (95% CI: 15.02-20.25) months. Out of 45 patients with BMs, 28 (62.2%) progressed, whereas 70 of 88 patients without BMs (79.5%) progressed.

**Figure 2:**
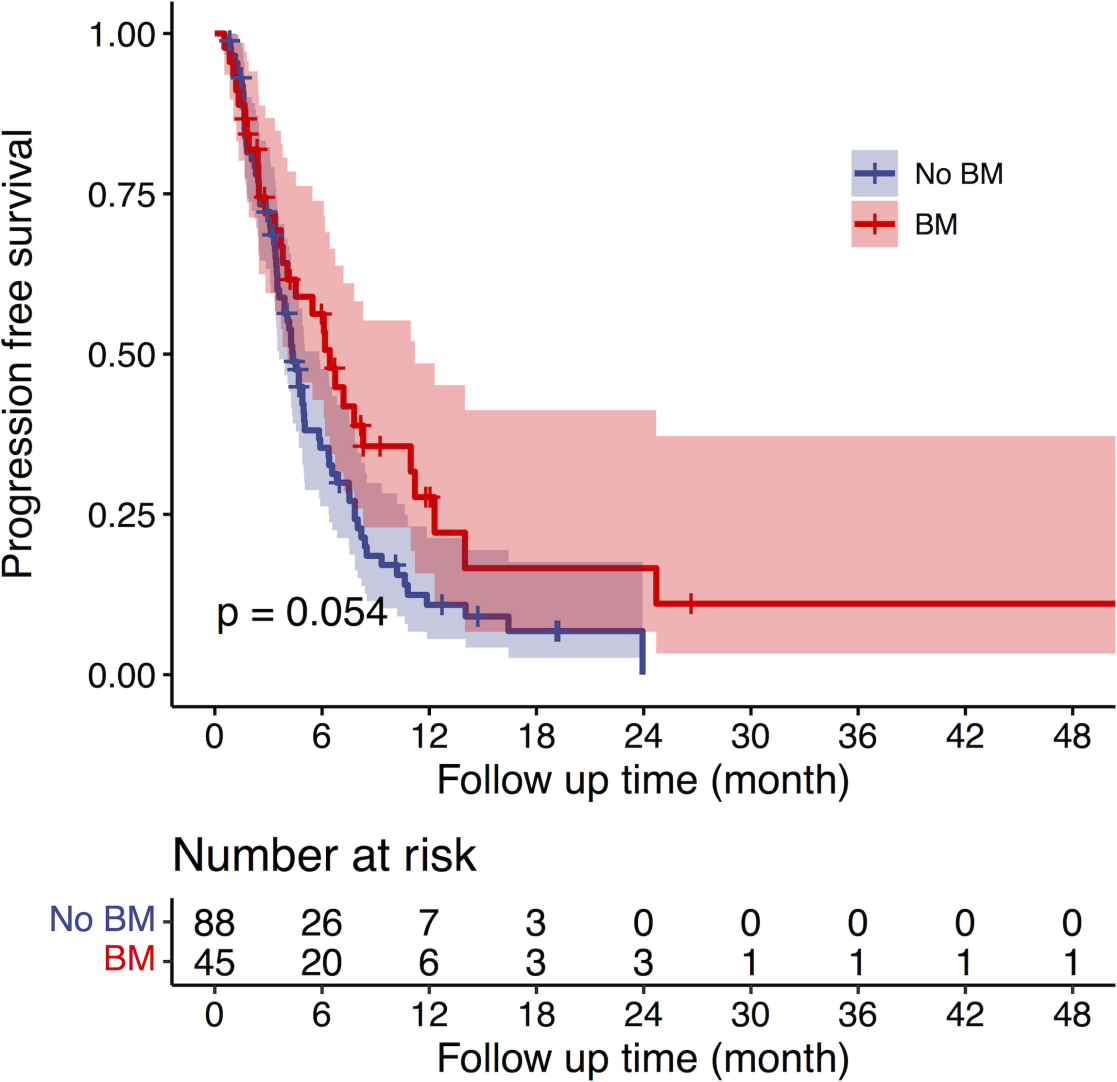
The median PFS times for patients with and without BMs were 6.43 months (95% CI: 4.70-8.17) and 4.37 months (95% CI: 3.71-5.04), respectively (*p* =0.054).

The median PFS times for patients with and without BMs were 6.43 months (95% CI: 4.70-8.17) and 4.37 months (95% CI: 3.71-5.04), respectively (*p* =0.054) (**Figure 2**). In univariable analysis for PFS, the status of extracranial metastases, treatment line of ICI and BM status had a *p*<0.1. In multivariable analysis, first-line treatment (*p*=0.000) was the only factor associated with improved PFS (**Table 2**). In patients who were treatment-naive, the median PFS for patients without and with BM were 8.83 months (95% CI 4.24-9.41) and 7.20 months (95% CI 5.38-9.02) (*p*=0.703).

**Table 2:** Univariable and Multivariable Analyses of Covariables Associated With Progression-Free Survival.

Variable	Univariable analysis		Multivariable analysis	
	HR (95% CI)	*p*	HR (95% CI)	*p*
Age (years)				
>60 vs. <50	1.333 (0.689-2.580)	0.394		
50-60 vs. <50	0.918 (0.469-1.800)	0.804		
Gender, male vs. female	0.958 (0.544-1.686)	0.881		
KPS, <90 vs. ≥90	1.284 (0.864-1.908)	0.217		
Smoking history, no vs. yes	1.305 (0.833-2.045)	0.245		
Extracranial metastases at start of treatment, no vs. yes	0.452 (0.217-0.945)	0.035	0.531 (0.235-1.200)	0.128
ICI inhibitor, PD-1 vs. PD-L1	1.029 (0.650-1.630)	0.903		
Line of ICI treatment, 1^st^ line vs. 2^nd^ and more	0.542 (0.364-0.807)	0.003	0.472 (0.313-0.711)	0.000
Thoracic RT before start of ICI treatment, no vs. yes	0.845 (0.565-1.264)	0.845		
Brain RT before start of ICI treatment, no vs. yes	0.879 (0.548-1.410)	0.594		
BM status, no vs. yes	1.524 (0.988-2.352)	0.057	1.509 (0.922-2.468)	0.101

KPS, Karnofsky Performance Score; BM, brain metastasis; RT, radiation therapy; ICI, immune checkpoint inhibitor; PD-1, programmed cell death 1; PD-L1, programmed cell death ligand 1; HR: hazard ratio; K-M, Kaplan−Meier.

After initiation of ICI therapy, three patients (3.4%) in the without-BM group received PCI and 13 (28.9%) patients in the with-BM group received brain radiotherapy. The numbers of patients of first-site progression with intracranial-only, extracranial-only, both sites, and no failure in the without-BM group were 7 (8.0%), 59 (67.0%), 4 (4.5) and 18 (20.5%), respectively. The corresponding numbers in the BM group were 7 (15.6%), 20 (44.4%), 1 (2.2) and 17 (37.8%) (*p*=0.039, **Figure 1**, **Supplementary Table 2A**). The corresponding numbers based on whether patients received the brain radiotherapy or not were reported in **Supplementary Table 2B**. In total, 80 patients in the without BM group and 9 patients in the with BM group never received brain radiation and the failure pattern was shown in **Supplementary Table 2C**. In this subgroup of patients, 6.3% (5/80) in without BM group and 22.2% (2/9) in with BM group had intracranial failure as first-site progression.

**Figure 3:**
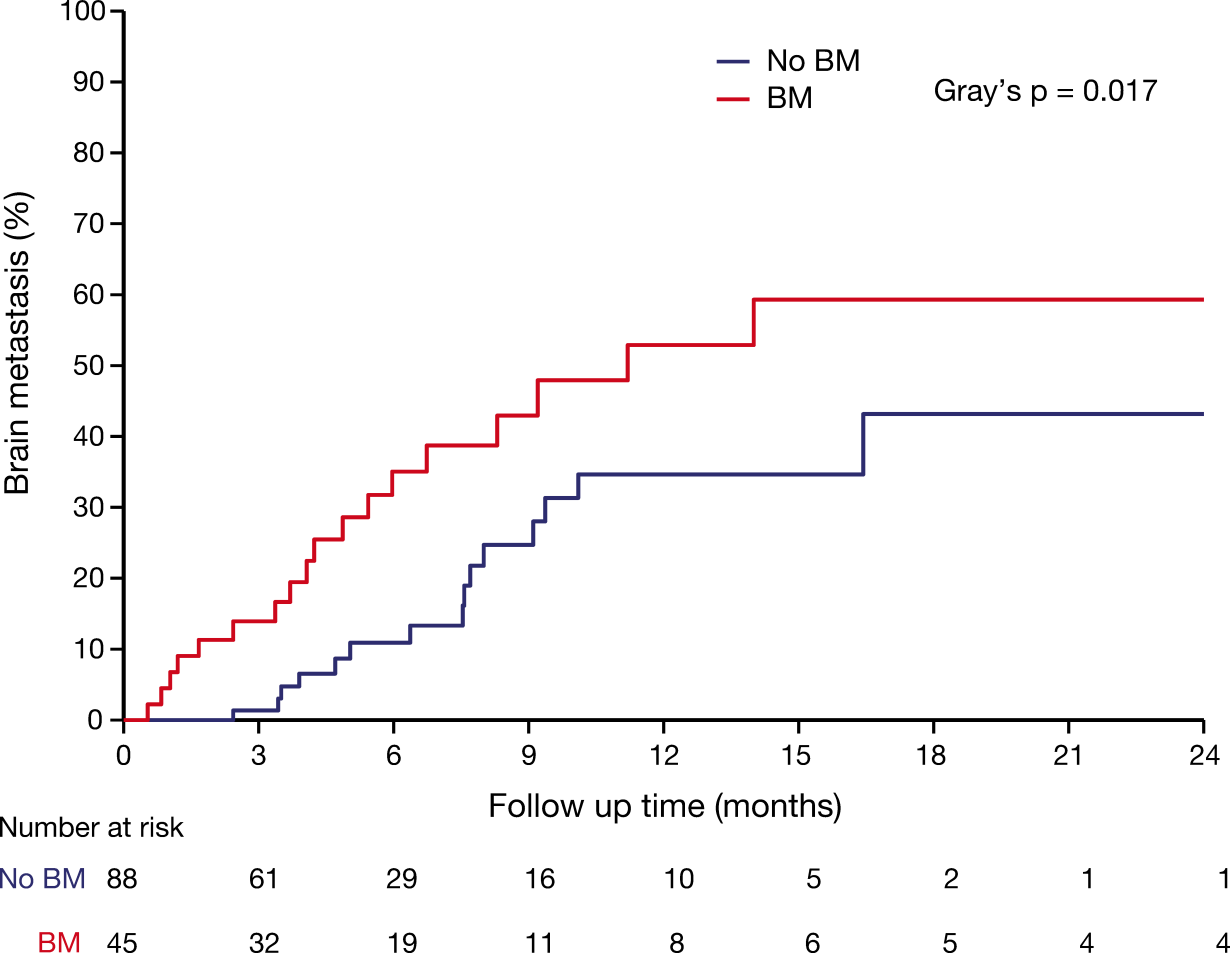
Cumulative incidence of intracranial progression using competing risks regression analysis in patients with BM and without BM.

At the last follow-up, 15 (17.0%) patients in the without-BM group and 19 (42.2%) patients in the BM group experienced intracranial failure. The cumulative incidences of brain metastases at 6, 12, and 18 months were 10.9% (95% CI: 4.31–21.1), 34.7% (19.2–50.0), and 43.2% (21.7–63.0) in the without-BM group and 35.0% (19.7–50.8), 52.9% (31.2–70.1), and 59.3% (35.7–76.7), respectively, in the BM group (Gray’s *p*=0.017; **Figure 3**). The post progression treatments after intracranial cancer progression in patients with BM and without BM were detailed in **Supplementary Table 3**.

#### 3.2.3 Overall survival

Of the 45 patients with BMs, 16 (35.6%) passed away, whereas 48 of 88 patients without BMs (54.5%) died. The median OS times for patients with and without BMs were 31.43 months (95% CI: 6.51-56.35) and 13.37 months (95% CI: 9.37-17.37), respectively (*p* =0.033). In univariable analysis for OS, age, KPS score, status of extracranial metastases, treatment line of ICI and BM status had a *p*<0.1. In multivariable analysis, patients who were younger and treatment-naive were associated with an improved OS (**Supplementary Table 4**). In patients who were treatment-naive, the median OS for patients without and with BM were 21.73 months (95% CI 12.60-30.86) and not reached (*p*=0.061).

## 4 Discussion

Immunotherapy has shown increasing potential and power in cancer treatment (15). In this retrospective cohort of patients with extensive-stage SCLC, 45 (33.8%) had brain metastases at the start of ICI treatment, while the BM incidence rates from prospective and randomized studies ranged from 8.7% to 12.1% (5–7). The ORR and DCR of patients with BMs and without BMs did not show significant differences. The ORR in the treatment-naive patients in our cohort was comparable to the data in clinical studies that only included patients with stable BMs, which ranged from 60.2% to 67.9% in the ICI+chemotherapy group (**Supplementary Table 5**). The PFS data in these trials ranged from 5.1 to 5.2 months (5, 6). For patients with BMs, the ORR and DCR were not different between patients with active BMs and those with stable BMs. Our data showed that five patients had a dissociated intracranial and extracranial response, one patient had brain-only PD with an extracranial response and six patients had only extracranial PD with intracranial stability, which indicates that the brain is not a shelter for cancer cells under ICI treatment. More clinical studies of immunotherapy for SCLC with brain metastases are warranted (**Supplementary Table 6**).

The data from NSCLC clinical trials demonstrated the same conclusion. A retrospective study showed that the intracranial and extracranial response rates were not different in less selected patients when treated with ICIs. The most recent prospective clinical trial aiming to demonstrate the efficacy of ICIs in BMs concluded that BM lesions responded to ICI treatment as extracranial lesions did (13). Even though the results are in contrast to the traditional theory that antibodies cannot cross the blood-brain barrier, the data showed that the existence of the blood-tumor barrier, a channel for antibodies and immune cells, may shed some light on the reason that immunosuppressive factors can reach cancer cells in the brain (16–18). In addition, a study demonstrated that ICIs passed through the barrier with CD8+ T cells as active transportation (19). So the ICIs could break the immunosuppressive microenvironment raised by the metastatic tumor cells. What’s more, the ICI treatment may prevent the homing of metastatic cells to the perivascular space in the brain and stop new sites of metastasis growing, which further explained why the intracranial-only first-site progression are few both in with-BM and without-BM patients(20, 21).

BM failure rate and timing are crucial due to their indicative roles for the doctor when prescribing radiotherapy for the brain, PCI or other options. Our data showed that patients had different failure patterns between groups, and only 7 patients (8.0%) without BMs and 7 patients (15.6%) with BMs had intracranial failure only as the first site of progression, which accounted for much less than those with extracranial failure and those with both (71.6% and 46.6%, respectively), given 10.0% (8/80) and 80% (36/45) of patients in without-BM and BM group received brain radiotherapy (previously or concurrently). In patients who never received brain radiation, the intracranial failure were 6.3% in the without-BM group and 22.2% in the with-BM group, which indicated that intracranial failure is usually not an indication to change the systemic therapy.

In the pre-ICI era, the role of PCI in ES-SCLC is equivocal. Some pivotal studies have demonstrated that it lowers intracranial failure and prolongs overall survival (22–24). However, a more contemporary study reported by Dr. Takahashi et al. based on MR surveillance showed that PCI did not improve the BM failure rate and survival (25). In the ICI era, there was no evidence or clinical data indicating the use of PCI, and the guidelines are conservative on the recommendation of PCI. In our study, in the without-BM group, the cumulative incidences of brain metastases at 6, 12, and 18 months were 15.0% (95% CI: 9.2–22.3), 32.9% (24.3–41.7), and 40.1% (31.0–49.1), respectively, which is much lower than the chemo alone group (12 months: 40.4-59.0%) and comparable to the chemo+PCI group (12 months: 14.6%-32.9%) in the pivotal trial of PCI (23, 25, 26). This suggests that patients who are responsive to ICI+chemo may be free from PCI. However, whether PCI further decreases the BM failure rate or prolongs OS remains to be determined.

Although our study demonstrated that BMs are not associated with poorer response and survival, several limitations should be taken into account. First, this is a retrospective study that has inherent biases and unstandardized follow-up despite our efforts to narrow our inclusion criteria. In this study, the age of patients, extracranial tumor burden, and radiation history (thoracic or brain radiotherapy previously) were not balanced between groups. Second, we failed to analyze the effect of corticosteroid use on the ORR of patients with BMs under the theory that corticosteroids would decrease the efficacy of ICIs, even though 5 patients (11.1%) in our BM cohort received dexamethasone due to symptomatic BMs or other reasons. Third, we pooled all patients and compared PFS and OS in a combination of patients with recurrence and newly diagnosed disease. Finally, we did not perform multivariate analysis of PFS and OS in the BM subgroup due to its small number of patients, and it is of interest for future research.

## 5 Conclusion

In conclusion, the ORR and DCR of patients with BMs and without BMs did not show significant differences. The failure patterns between groups were different, with few patients in both groups first experiencing intracranial progression only. Although patients with BMs progressed more often in the brain than did patients without preexisting BMs, the presence of BMs was not significantly associated with a poorer PFS and OS with ICI treatment in multivariate analysis.

## Data availability statement

The raw data supporting the conclusions of this article will be made available by the authors, without undue reservation.

## Ethics statement

The studies involving human participants were reviewed and approved by the institutional review board of Chinese People’s Liberation Army (PLA) General Hospital. Written informed consent for participation was not required for this study in accordance with the national legislation and the institutional requirements.

## Author contributions

CW: Conceptualization, Methodology, Formal analysis, Writing- Original draft preparation. SM: Data curation, Writing- Reviewing and Editing. XY: Data curation, Writing- Reviewing and Editing. LL: Data curation, Writing- Reviewing and Editing. HT: Data curation, Writing- Reviewing and Editing. FZ: Data curation, Writing- Reviewing and Editing. RL: Data curation, Writing- Reviewing and Editing. YH: Conceptualization, Methodology, Writing- Reviewing and Editing. LW: Conceptualization, Methodology, Writing- Reviewing and Editing. All authors contributed to the article and approved the submitted version.
