# Supplementary material for: Outcome of immune checkpoint inhibitors in patients with extensive-stage small-cell lung cancer and brain metastases
Source: Front Oncol. 2023 May 5;13:1110949. doi: 10.3389/fonc.2023.1110949 (PMC10196483; doi:10.3389/fonc.2023.1110949)
Supplement: Supplementary file 1 [file DataSheet_1.docx]

| Supplementary Table 1. Patient Characteristics with BM | | | | |
| --- | --- | --- | --- | --- |
|  | Patients with Baseline Brain Metastases （N=45)(%) | Patients with Active Baseline Brain Metastases (N=25)(%) | Patients with Stable Baseline Brain Metastases (N=20)(%) | *p* |
| Smoking history |  |  |  |  |
| No | 10 (22.2) | 5 (20.0) | 5 (25.0) | 0.731 |
| Yes | 35 (77.8) | 20 (80.0) | 15 (75.0) |  |
| Gender |  |  |  |  |
| Male | 39 (86.7) | 22 (88.0) | 17 (85.0) | 1.000 |
| Female | 6 (13.3) | 3 (12.0) | 4 (15.0) |  |
| PD-1/PD-L1 inhibitor |  |  |  |  |
| PD-1 | 37 (82.2) | 22 (88.0) | 15 (75.0) | 0.435 |
| PD-L1 | 8 (17.8) | 3 (12.0) | 5 (25.0) |  |
| Line of ICI treatment |  |  |  |  |
| 1 | 15 (33.3) | 13 (52.0) | 2 (10.0) | 0.012 |
| 2 | 16 (35.6) | 7 (28.0) | 9 (45.0) |  |
| Over 2 | 14 (31.1) | 5 (20.0) | 9 (45.0) |  |
| Largest size of BM |  |  |  |  |
| ≤10 mm | 18 (40.0) | 9 (36.0) | 9 (45.0) | 0.760 |
| >10mm | 27(60.0) | 16 (64.0) | 11 (55.0) |  |
| Numbers of BM |  |  |  |  |
| Median (range) | 3 (1-13) | 4 (1-13) | 2 (1-10) | 0.007 |
| Disease-specific GPA |  |  |  |  |
| 0-1 | 10 (22.2) | 7 (28.0) | 3 (15.0) | 0.620 |
| 1.5-2.0 | 19 (42.2) | 11 (44.0) | 8 (40.0) |  |
| 2.5-3.0 | 12 (26.7) | 5 (20.0) | 7 (35.0) |  |
| 3.5-4.0 | 4 (8.9) | 2 (8.0) | 2 (10.0) |  |
| Symptom from BM |  |  |  |  |
| No | 43 (95.6) | 24 (96.0) | 19 (95.0) | 1.000 |
| Yes | 2 (4.4) | 1 (4.0) | 1 (5.0) |  |
| Brain RT before start of ICI treatment |  |  |  |  |
| No | 21 (46.7) | 19 (76.0) | 2 (10.0) | 0.000 |
| Yes | 24 (53.3) | 6 (24.0) | 18 (90.0) |  |
| Abbreviations: KPS, Karnofsky Performance Score; BM, brain metastasis; GPA, graded prognostic assessment; RT,radiation therapy; ICI: immune checkpoint inhibitor;PD-1:programmed cell death 1; PD-L1:programmed cell death ligand 1 | | | | |

| Supplementary Table 2a. First site of progression by brain metastases status. | | |  |
| --- | --- | --- | --- |
|  | Patients without Baseline Brain Metastases (N=88)(%) | Patients with Baseline Brain Metastases （N=45)(%) | *p* |
| First site of progression |  |  | 0.039 |
| Intra-cranial alone | 7 (8.0) | 7 (15.6) |  |
| Extra-cranial alone | 59 (67.0) | 20 (44.4) |  |
| Both | 4 (4.5) | 1 (2.2) |  |
| No failure | 18 (20.5) | 17 (37.8) |  |

| Supplementary Table 2b. First site of progression by brain radiotherapy status. | | |  |
| --- | --- | --- | --- |
|  | No brain radiotherapy (N=89)(%) | Had brain radiotherapy before ICI or with ICI (N=44)(%) | *p* |
| First site of progression |  |  | 0.208 |
| Intra-cranial alone | 7 (7.9) | 7 (15.9) |  |
| Extracranial alone | 58 (65.2) | 21(47.7) |  |
| Both | 3 (3.4) | 2 (4.5) |  |
| No failure | 21 (23.6) | 14 (31.8) |  |

| Supplementary Table 2c. First site of progression in patients never received brain radiation by brain metastases status | | |  |
| --- | --- | --- | --- |
|  | Patients without Baseline Brain Metastases (N=80)(%) | Patients with Baseline Brain Metastases (N=9)(%) |  |
| First site of progression |  |  |  |
| Intra-cranial alone | 5 (6.3) | 2 (22.2) |  |
| Extracranial alone | 56 (70.0) | 2 (22.2) |  |
| Both | 2 (2.5) | 1 (11.1) |  |
| No failure | 17(21.3) | 4 (44.4) |  |

| Supplementary Table 3. The post-progression treatments after intracranial cancer progression in patients with BM and without BM. | | | | |
| --- | --- | --- | --- | --- |
|  | Total Population had intracranial progression (N=34) | Patients without Baseline Brain Metastases (N=15) | Patients with Baseline Brain Metastases (N=19) |  |
| Continue ICIs+brain RT | 11 | 7 | 4 |  |
| Continue ICIs+other systemic therapy | 1 | 1 | 0 |  |
| Other systemic therapy+brain RT | 4 | 3 | 1 |  |
| Other systemic therapy | 8 | 0 | 8 |  |
| Best supportive care | 10 | 4 | 6 |  |
| Abbreviations: RT, radiation therapy | | | |  |

| Supplementary Table 4. Univariable and Multivariable Analyses of Covariables Associated With Overall Survival | | | | |
| --- | --- | --- | --- | --- |
| Variable | Univaraible analysis | | Multivariable analysis |  |
|  | HR (95%CI) | *p* | HR (95%CI) | *p* |
| Age at BM (years) |  |  |  |  |
| >60 vs <50 | 4.617(1.415-15.071) | 0.011 | 4.013(1.223-13.166) | 0.022 |
| 50-60 vs <50 | 2.331(0.699-7.778) | 0.169 | 2.583 (0.770-8.662) | 0.124 |
| Gender, male vs female | 1.789(0.722-4.150) | 0.175 |  |  |
| KPS, <90 vs ≥90 | 1.691(1.034-2.766) | 0.036 | 1.580 (0.960-2.600) | 0.072 |
| Smoking history, no vs yes | 0.590(0.308-1.131) | 0.112 |  |  |
| Extra-cranial metastases at start of treatment, no vs yes | 0.399(0.145-1.100) | 0.076 | 0.581 (0.187-1.811) | 0.35 |
| ICI inhibitor, PD-1 vs PD-L1 | 1.052(0.569-1.946) | 0.871 |  |  |
| Line of ICI treatment, 1 vs more | 0.464(0.269-0.800) | 0.006 | 0.464 (0.264-0.814) | 0.007 |
| Thoracic RT before start of ICI treatment, no vs yes | 0.693 (0.423-1.135) | 0.145 |  |  |
| Brain RT before start of ICI treatment, no vs yes | 1.248 (0698-2.326) | 0.421 |  |  |
| BM status, no vs yes | 1.844(1.043-3.260) | 0.035 | 1.813 (0.940-3.498) | 0.076 |

| Supplementary Table 5. Study characteristics of published prospective clinical trials and our research | | | | | |
| --- | --- | --- | --- | --- | --- |
| Reference | Number of patients treated with ICI+chemotherapy | Number of patients with BM (percent) | ORR | DCR | Median PFS |
| Paz-Ares et al., 2019 | 268 | 28 (10.4%) | 67.91% | 75.37% | 5.1 months (95% CI 4·7–6·2) |
| Horn et al., 2018 | 201 | 17(8.5%) | 60.20% | 81.10% | 5.2 months (95% CI, 4.4–5.6) |
| [Rudin et al., 2020,](#RANGE!#REF!) | 228 | 3314.5%) | 70.60% | 88.10% | 4.5 months (95% CI, 4.3-5.4) |
| This research, whole cohort | 133 | 45(33.8%) | 48.12% | 79.70% | 4.7 months (95% CI, 4.0-5.4) |
| This research, patients with no prior treatment | 56 | 15(26.8%) | 80.35% | 94.60% | 7.2 months (95%CI, 5.4-9.0) |
| Abbreviations: ORR, objective response rate; DCR, disease control rate; PFS, progression free survival. | | | | | |

Reference:

PAZ-ARES, L., DVORKIN, M., CHEN, Y., REINMUTH, N., HOTTA, K., TRUKHIN, D., STATSENKO, G., HOCHMAIR, M. J., ÖZGÜROĞLU, M., JI, J. H., VOITKO, O., POLTORATSKIY, A., PONCE, S., VERDERAME, F., HAVEL, L., BONDARENKO, I., KAZARNOWICZ, A., LOSONCZY, G., CONEV, N. V., ARMSTRONG, J., BYRNE, N., SHIRE, N., JIANG, H. & GOLDMAN, J. W. 2019. Durvalumab plus platinum-etoposide versus platinum-etoposide in first-line treatment of extensive-stage small-cell lung cancer (CASPIAN): a randomised, controlled, open-label, phase 3 trial. *Lancet,* 394**,** 1929-1939.

HORN, L., MANSFIELD, A. S., SZCZĘSNA, A., HAVEL, L., KRZAKOWSKI, M., HOCHMAIR, M. J., HUEMER, F., LOSONCZY, G., JOHNSON, M. L., NISHIO, M., RECK, M., MOK, T., LAM, S., SHAMES, D. S., LIU, J., DING, B., LOPEZ-CHAVEZ, A., KABBINAVAR, F., LIN, W., SANDLER, A. & LIU, S. V. 2018. First-Line Atezolizumab plus Chemotherapy in Extensive-Stage Small-Cell Lung Cancer. *N Engl J Med,* 379**,** 2220-2229.

RUDIN, C. M., AWAD, M. M., NAVARRO, A., GOTTFRIED, M., PETERS, S., CSŐSZI, T., CHEEMA, P. K., RODRIGUEZ-ABREU, D., WOLLNER, M., YANG, J. C., MAZIERES, J., ORLANDI, F. J., LUFT, A., GÜMÜŞ, M., KATO, T., KALEMKERIAN, G. P., LUO, Y., EBIANA, V., PIETANZA, M. C. & KIM, H. R. 2020. Pembrolizumab or Placebo Plus Etoposide and Platinum as First-Line Therapy for Extensive-Stage Small-Cell Lung Cancer: Randomized, Double-Blind, Phase III KEYNOTE-604 Study. *J Clin Oncol,* 38**,** 2369-2379.

| Supplementary Table 6. The clinical studies of immunotherapy for SCLC with brain metastases. | | | | | | |
| --- | --- | --- | --- | --- | --- | --- |
| Included patients | Phase | Treatment | Endpoint/Results | (planned) Sample size | Clinical trial number/PMID | Status |
| Untreated ES-SCLC with asymptomatic BMs. | Phase 2 | Atezolizumab+EC | 2 year of intracranial PFS | 60 | NCT04610684 | Active |
| Presenting with previously un-irradiated BMs | Phase 2 | Nivolumab+Radiosurgery | 1 year of intracranial PFS | 26 | NCT02978404 | Active, not recruiting |
| ES-SCLC with BMs | Retrospective study | anti-PD-L1+brain radiotherapy | median OS : not reached; median PFS: 9.4 months;  intracranial PFS :8.2 months ORR: 73.3% | 15 | PMID: 35976547 | Published |
| Abbreviations: ES-SCLC, extensive stage; BMs. brain metastases; EC, Etoposide+Carboplatin; PFS, progression free survival; OS, overall survival; ORR, Objective response rate; PD-L1, programmed cell death ligand 1. | | | | | | |
